# An improved method for efficient recovery of high quality DNA from date palm (*Phoenix dactylifera* L; *Arecaceae*)

**DOI:** 10.1016/j.mex.2021.101384

**Published:** 2021-05-21

**Authors:** M.I.S. Safeena, Y. Dissanayake, M.C.M. Zakeel, L. Warnakula, R. Cooray, D.A.R.K. Dayarathna

**Affiliations:** a Department of Biological Science, Faculty of Applied Sciences, South Eastern University of Sri Lanka, Sammanthurai, Sri Lanka; b Section of Genetics, Institute for Research and Development, Colombo, Sri Lanka; c Department of Plant Sciences, Faculty of Agriculture, Rajarata University of Sri Lanka, Puliyankulama, Anuradhapura, Sri Lanka; d The University of Queensland, Queensland Alliance for Agriculture and Food Innovation, Centre for Horticultural Science, GPO Box 267, Brisbane, QLD 4001, Australia

**Keywords:** *Phoenix dactylifera*, DNA extraction methods, Contaminants free DNA, Cetyltrimethylammoniumbromide, Improved CTAB method

## Abstract

Date palm (*Phoenix dactylifera* L; *Arecaceae*) is one of a few fruit trees that can remarkably grow in dessert agroecosystems that are characterized by extreme temperature fluctuations. Due to increasing demands for dates in the global market and commercial cultivation in many countries, the tree is currently under extensive research in many countries, particularly to improve the germplasm using different molecular tools. Most molecular techniques largely depend on good quality DNA in significant quantities, which are highly compromised by the presence of various contaminants in DNA. The traditional cetyltrimethylammoniumbromide (CTAB) based method has failed to produce good quality DNA from date palm due to hard fibrous nature of tissue. On the basis of previous studies, commercial DNA extraction kits are not economical although they are very effective. Therefore, we have developed an improved DNA extraction protocol by modifying the original CTAB method to produce extra pure DNA in large quantities. The novel method has been validated using different quality testing approaches. This cost-effective method can be used successfully for DNA extraction from date palm. Moreover, this improved method may have potential for DNA extraction from other palms that have similar leaf texture to date palm leaves, but this method needs to be tested for other palms before being used. The improved method has following key modifications:

• Grinding of plant tissue in liquid nitrogen and subsequent lysis of cells in CTAB buffer that has increased concentration of ß-mercaptoethanol
• Repeated steps of chloroform: IAA extraction and ethanol washing
• Addition of RNase A before the DNA precipitation step

## Graphical Abstract

**Figure:**
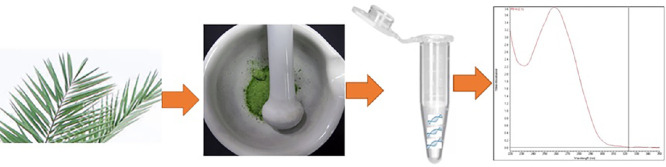

**Table:** Specifications Table

**Subject Area**	Biochemistry, Genetics and Molecular Biology
**More specific subject area**	Plant molecular biology, DNA extraction
**Method name**	Novel method of DNA extraction
**Name and reference of original method**	Original CTAB protocol J.J. Doyle, J.L. Doyle, A rapid DNA isolation procedure for small quantities of fresh leaf tissue, Phytochemical Bulletin 19 (1987) 11-15.
**Resource availability**	*n/a*

## Background

The success of many molecular investigations strongly depend on the extraction of high quality DNA in considerable quantities. The purity and good quantity of DNA are vital for most molecular experiments including PCR amplification, restriction digestion and DNA sequencing, and also for many downstream processes [1]. Polyphenols, polysaccharides and RNA are the main contaminants of DNA that can mostly interfere with the downstream assays [2], [3], [4]. The presence of these contaminants results in poor quality DNA that remains the most common reason for failures in PCR reactions and other molecular assays [5]. The co-extraction of these contaminants with DNA is a common problem in trees including date palm than most other plants [6].

Date palm (*Phoenix dactylifera* L) is a monocotyledonous, dioecious, perennial fruit tree belonging to family Arecaceae. It is one of the most ancient plants distributed in different parts of the world mainly (~90%) in the Middle East and North Africa region [7,8]. In Sri Lanka, the date palm is distributed in the coastal belt spanning from the northeast to the southeastern regions. Moreover, sporadic distribution of date palms can also be seen in the coastal regions of the North Western and Western provinces. Sex identification of immature plants is a major challenge to the date palm industry in order to avoid the unnecessary management of male trees before until they attain sexual maturity that generally takes 5 − 10 years [9,10]. In recent years, the early identification of gender specific DNA sequences efficiently addressed this issue. For this molecular assay, extraction of good quality DNA is vital.

The traditional cetyltrimethylammoniumbromide (CTAB) based method does not help with DNA extraction from date palms, mainly because their leaves are rigid and fibrous, which render the manual grinding of immature leaves with CTAB buffer using a mortar and pestle very difficult [1]. Gradient centrifugation using CsCl is a useful step to produce high quality DNA [11]; this, however, is a lengthy procedure and not preferred for studies with a large number of samples. Commercial DNA extraction kits have been validated for date palm [12], but they are not cost effective. Currently available DNA extraction protocols for date palm and various ‘ready-to-use’ commercial kits require liquid nitrogen for the crushing-into-powder step of plant materials to maximize the yield of DNA. Furthermore, these commercial extraction kits are relatively expensive compared to the original method described by Doyle and Doyle [13], particularly when DNA extraction from a large number of samples is needed for a series of molecular experiments. Therefore, this study was aimed to develop a simple, efficient and improved protocol for the extraction of extra pure DNA in satisfactory concentrations from immature date palm leaves. The original CTAB method [13] was successfully modified to develop the improved protocol for efficient extraction of DNA from date palm germplasm in Sri Lanka.

**Fig. 1:**
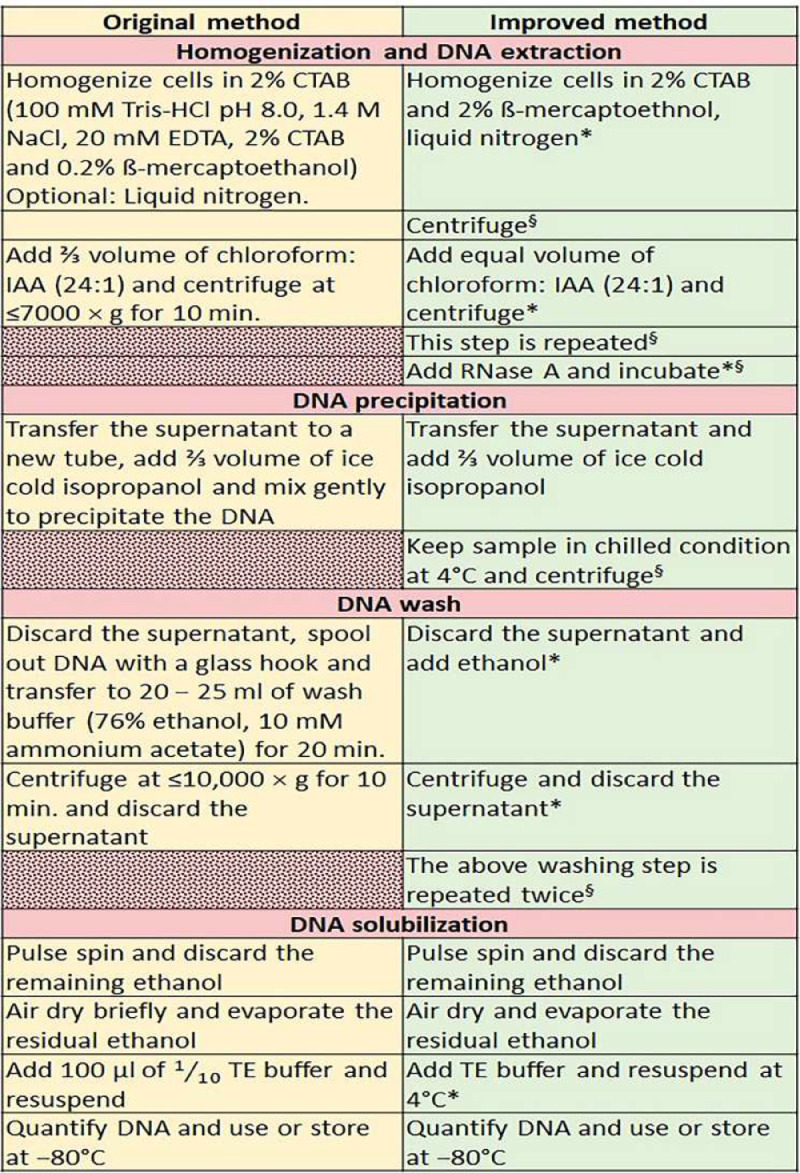
The comparison of improved DNA extraction protocol with the original method by Doyle and Doyle [13]. The red patterned fill indicates the additional steps that included in the improved method but not available in the original method, * indicates modified step and **§** indicates new/entirely modified step.

The novel protocol is featured by the addition of some extra steps and increased quantity of additives to the extraction buffers (Fig. 1). For instance, the improved method has an additional chloroform: isoamyl alcohol (IAA) phase separation step. Fig. 1 summarizes the steps of the original and the improved methods. It was evident from the validation of the improved method that the quality and quantity of extracted DNA were significantly improved by the new protocol. The only limitation of the novel method is that the addition of a few extra steps has lengthened the time required for the whole process of DNA extraction and the extra time needed does not generally exceed 30 – 35 minutes per batch. However, experienced investigators can easily shorten this duration when adequate reagents and necessary equipment are available.

## Detailed procedure of improved method

### Required reagents and equipment

CTAB, Tris-HCl, EDTA, RNase, chloroform, isopropanol and ethanol (EtOH) (all reagents were from Sigma-Aldrich). 1.5 and 2 ml safe-lock microcentrifuge tubes, micropipettes, micropipette tips, refrigerated microcentrifuge and Nanodrop^TM^ 2000 spectrophotometer.

### Homogenization and DNA extraction

1. Grind leaf tissue into powder using liquid nitrogen and a mortar and pestle.*
2. Homogenize cells in 2% CTAB buffer (1 M Tris-HCl pH 8.0, 5 M NaCl, 0.5 M EDTA, 2% CTAB and 2% ß-mercaptoethnol).* (all constituents of buffer is increased except the concentration of CTAB)
3. Centrifuge at 4°C ≤12,000 × g for 5 minutes and collect the supernatant.**^§^**
4. Add equal volume of chloroform: IAA (24:1) to the supernatant, centrifuge at 4°C ≤10, 000 × g for 10 min and collect the supernatant.*
5. Repeat the step 4 again.**^§^**
6. Add 3 µl of RNase A (10 µg/ml) to the supernatant recovered in step 5 and incubate at 37°C for one hour (an optional step performed at the end of the procedure in the original method).*

***DNA precipitation***

7. Transfer the above RNase treated mixture to a new 1.5 ml microcentrifuge tube containing ⅔ volume of ice-cold isopropanol.
8. Incubate the mixture at 4°C for 20 – 40 min.**^§^**
9. Precipitate the DNA by centrifuging at 4°C ≤12,000 × g for 10 min.

*At this time, the DNA should be visible as a small white pellet. Nevertheless, the less amount of DNA in the solution may not yield distinct white pellet.*

***DNA wash***

10. Remove and discard the supernatant.*
11. Add 150 µl of 75% EtOH.
12. Centrifuge at 4°C ≤14,000 × g for 10 min.*
13. Remove and discard the supernatant.*
14. Repeat this step further two times.**^§^**

a. Add 150 µl of 75% EtOH.
b. Centrifuge at 4°C ≤14,000 × g for 10 min.
c. Remove and discard the supernatant.
d. Add 150 µl of 75% EtOH.
e. Centrifuge at 4°C ≤14,000 × g for 10 min.
f. Remove and discard the supernatant.

*The supernatant can be removed by slightly sloping the tube carefully without collapsing the pellet.*

***DNA solubilization***

15. Pulse spin the pellet at room temperature and cautiously remove the remaining EtOH using a micropipette.
16. Airdry and evaporate the residual EtOH for 30 min (Alternatively, the residual EtOH can be removed by cautiously tilting the tube upside down onto a kimwipe tissue paper).
17. Add 20 – 30 µl of 1/10 TE buffer and resuspend the pellet at 4°C.*
18. Quantify the DNA and use or store at –80°C.

*Note:* * indicates modified step; **§** indicates new or entirely modified step

## Authentication of improved method

### Quality and quantity assessment

DNA was extracted from 33 randomly selected date palm trees using the original and the improved protocols separately. The purity and quantity of isolated DNA were determined using a Nanodrop^TM^ 2000 spectrophotometer. Optical density (OD) values at 230, 260 and 280 nm wavelengths, and A260/280 and A260/230 ratios were recorded. The integrity of the DNA was checked by agarose gel electrophoresis.

DNA concentration and the absorbance ratios of 260/280 and 260/230 were subjected to Levene's test for homogeneity of variance and Shapiro-Wilk normality tests in order to check if the data show homogeneity of variance and normal distribution. As data proved to be not normal, the non-parametric Kruskal-Wallis test was used evaluate the effect of the improved method of DNA extraction as opposed to the original method. All statistical analyses were performed in R statistical software environment using “agricolae”, “MASS” and “car” packages.

The DNA concentration, and 260/280 and 260/230 absorbance ratio data were not normally distributed (*P*<0.05) and showed heterogeneous variance (*P*<0.05) among the treatments (original and improved methods of DNA extraction). The DNA extraction methods had significant effects on DNA concentration as well as the quality parameters measured in terms of 260/280 and 60/230 ratios (*P*<0.05, Table 1).

**Table 1:** Kruskal-Wallis rank sum test of different DNA quantity and quality parameters for the original and improved DNA extraction methods for date palm (*Phoenix dactylifera* L.).

DNA quantity/quality parameters	χ^2^	df	*P*
Concentration (ng/µl)	44.26	1	<0.01
260/280 ratio	44.35	1	<0.01
260/230 ratio	44.55	1	<0.01

χ^2^: Chi-square value; df: degrees of freedom; *P*: probability

**Fig. 2:**
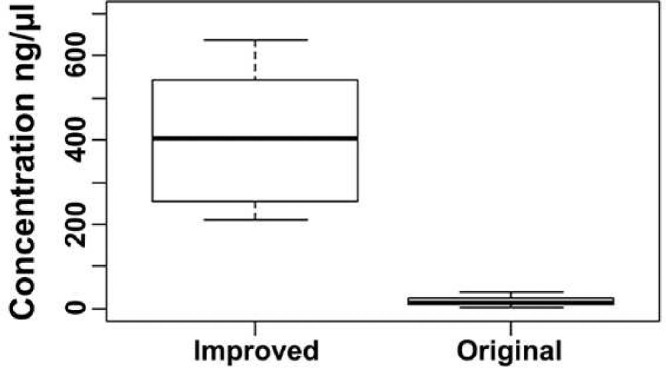
Box plots of DNA concentrations (ng/µl) based on the absorption spectra of DNA samples extracted using the original and improved methods of DNA extraction. Non-overlapping boxes indicate that the groups are different.

**Fig. 3:**
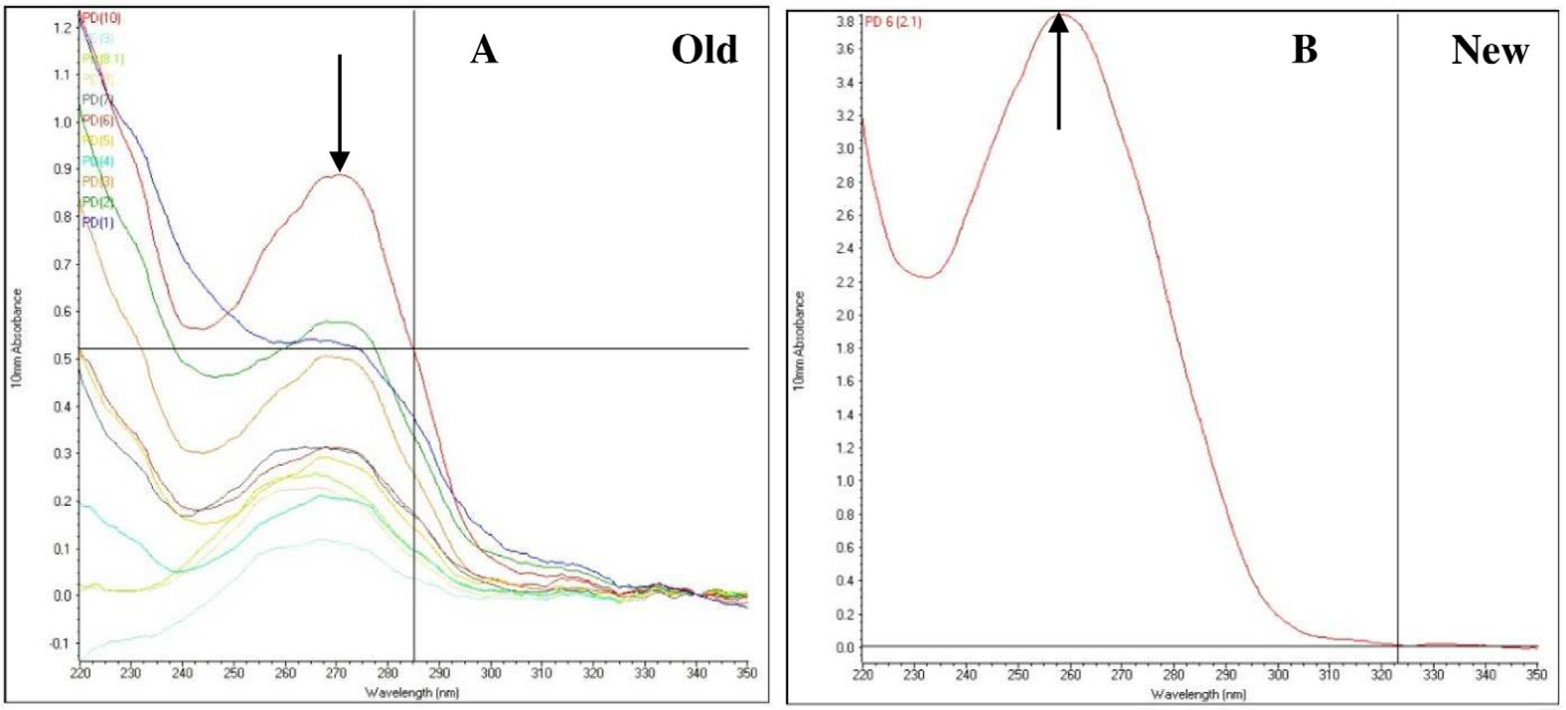
Representative spectra comparing the improved method (new) to the original DNA extraction method (old). The spectra were recorded using a NanoDrop 2000 spectrophotometer and overlayed for the comparison within and between group. The arrow appears over 270 nm in the original protocol (old - A) and 260 nm in the new protocol (new - B).

**Fig. 4:**
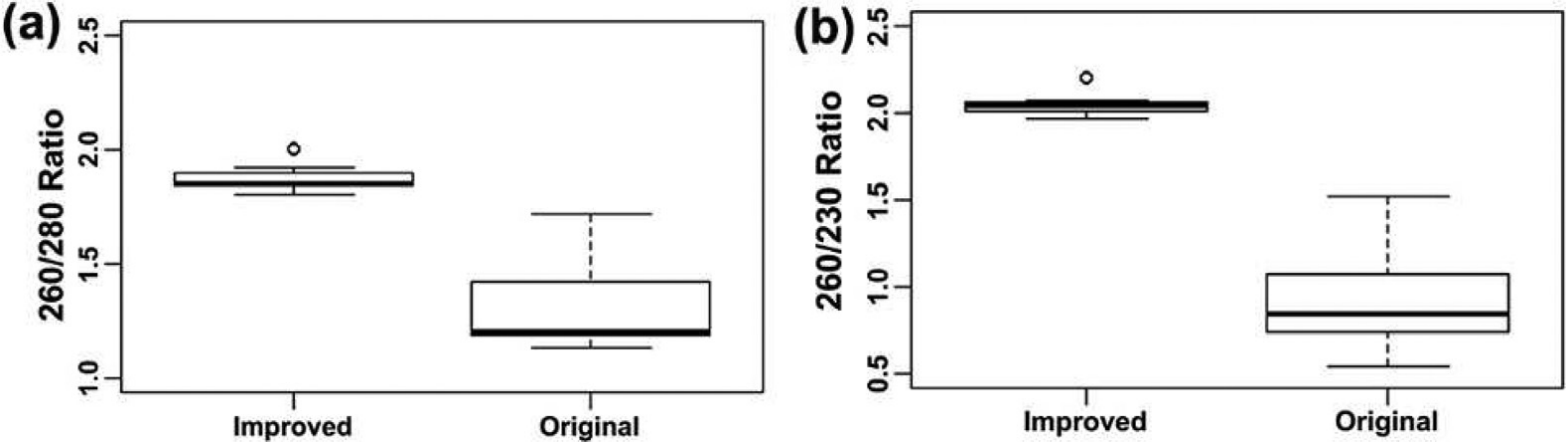
Box plot of 260/280 and 260/230 absorbance ratios (DNA quality parameters) for the original and improved methods of DNA extraction. Non-overlapping boxes indicate that the groups are different.

The results revealed that improved methods yielded significantly higher quantity of DNA (median = 400 ng/µl) compared with the original method (Fig. 2). The improved method showed a peak in absorbance at 260 nm indicating higher concentration of DNA and the value rapidly declined at 280 nm showing minimum level of contaminants (Fig. 3). However, the absorbance values at 260 and 280 nm of DNA samples extracted using the original method were not much different between them indicating low yield and the presence of high levels of contaminants (Fig. 3). Moreover, the 260/280 and 260/230 ratios of the improved method were very close to 1.8 and 2.0, respectively (Fig. 4). However, the 260/280 ratio of the original method showed a larger variance with a median value close to 1.2 (Fig. 4a). Similarly, the 260/230 ratio of the original method also had small values with a median of 0.75 (Fig. 4b). This shows that the improved method resulted in high yield of extra pure quality DNA as opposed to the original method.

The ratios of optical density (OD) at 260 nm and 280 nm measure the purity of DNA with respect to protein and other contaminants that absorb at 280 nm [14]. This ratio ranges between 1.7 and 2.0 for pure DNA and a value less than this range indicates presence of protein, phenol and other contaminants. The secondary measurement of pure DNA is the OD values at 260 nm and 230 nm in which salts absorb at 230 nm. Generally, the accepted value ranges between 2.0 and 2.2 for pure DNA. A value less than 1.8 indicates that the DNA has contaminants such as phenols, organic compounds, ethylenediamine tetraacetic acid (EDTA), carbohydrates and polysaccharides [1,15].

### Validation of quality and integrity of DNA using PCR

The quality and integrity of DNA was evaluated by a PCR reaction that amplified the *rbcL* gene (ribulose-1,5-bisphosphate carboxylase large chain) using RbclaF (5ꞌ-ATGCCACAAACAGAGACTAAAGC-3ꞌ) and RbclaR (5ꞌ-GTAAAATCAAGTCCACCRCG-3ꞌ) primer pairs [16,17]. The PCR was performed in a 20 µl reaction that contained 4 µl of 5 × FIREPol mastermix (Solis BioDyne, Estonia), 100 nM of each primer, and respective quantity of DNA template and nuclease free water to make up the volume. To bring the template DNA concentration to 25 ng/µl in each reaction, DNA from samples Pd23, Pd24, Pd29, Pd30 and Pd33 was added in volumes of 0.5, 1.0, 1.1, 1.2 and 0.8 µl, respectively. PCR conditions had an initial denaturation at 95°C for 5 min. and 33 cycles of denaturation at 95°C for 30 s, primer annealing at 55°C for 30 s and by extension at 72°C for 1 min, followed by final extension at 72°C for 5 min. PCR products were electrophoresed in a 0.8% agarose gel.

**Fig. 5:**
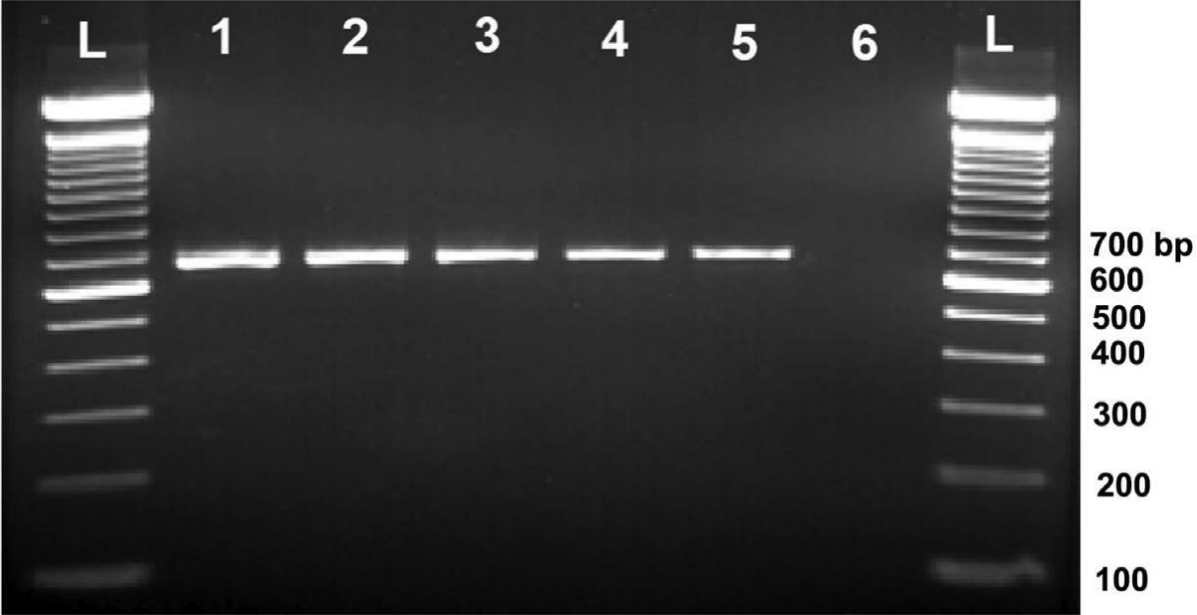
Agarose gel (0.8%) electrophoresis of PCR amplicons produced by rbcL primers with DNA samples extracted from date palm trees using the improved method of DNA extraction. L indicates 100 bp ladder; lanes 1 to 5 indicate DNA samples Pd23, Pd24, Pd29, Pd30 and Pd33, respectively; lane 6 indicates water (–ve control).

The results showed that DNA samples extracted by the novel improved methods presented good amplification as evidenced by clear and thick bands of expected amplicons in the gel indicating that the improved method resulted in good quality DNA in large quantities (Fig. 5). The PCR with DNA extracted using the original method resulted in a smear, indicating poor quality and integrity of DNA (data not shown). The negative control did not produce any amplicons (Fig. 5), confirming that there was not any cross contamination.

## Discussion and conclusion

DNA extraction from hard fibrous date palm leaves is tedious, particularly the grinding step of the tissues using a mortar and pestle and therefore time and labor consuming. Thus, liquid nitrogen can be used to make the grinding step more efficient. Immersing the pieces of plant leaf tissues in liquid nitrogen makes them brittle and eases the grinding of the tissues into a fine powder that facilitates the homogenization of cells with CTAB buffers [1,18]. Moreover, the ultra-low temperature of liquid nitrogen allows the tissues to remain frozen throughout the grinding step, thus curtailing enzymatic activities [19]. CTAB extraction buffer with the increased concentration of ß-mercaptoethanol, which is a strong reducing agent that can remove tannins and other polyphenolic compounds often present in crude plant extracts is possibly a factor that contributed to the extraction of contaminants-free good quality DNA in the improved method [20]. In contrast, Adams, Do and Ge-Lin [21] have shown that grinding of plant materials in ethanol enhances the precipitation of proteins including DNases. Furthermore, ß-mercaptoethanol that facilitates protein denaturation by breaking disulfide bonds between cysteine residues prevented protein contaminants in the DNA that was extracted by the improved method [22]. Two rounds of chloroform: IAA phase separation and subsequent centrifugation steps may have assisted in the separation and removal of cell debris and contaminants, thus increasing the quality of DNA. Moreover, the incubation of supernatant in ice-cold isopropanol at 4°C increased the quantity of DNA precipitation whereas repeated ethanol wash cleansed the DNA. Similar to the texture of date palm leaves, various other palms including *Areca* spp., *Borassus flabellifer, Calamus* spp. and *Caryota* spp. have hard, fibrous and difficult-to-grind leaf texture thus making the DNA extraction difficult [23,24]. Therefore, the improved DNA extraction protocol described in this study has potential for DNA extraction from other palm species, but the protocol should be tested for those species before being used for large-scale extraction and experiments.

In conclusion, this improved as well as cost-effective DNA extraction protocol may be implemented usefully to isolate good quality DNA in large quantity from date palm and may possibly tested for other palm species that have hard-to-grind fibrous leaf tissues and texture that is similar to date palm leaves.
